# Increased Adherence to Infection Control Practices Among Medical Laboratory Technicians During the COVID-19 Pandemic: A Self-Reported Survey Study

**DOI:** 10.5334/aogh.3378

**Published:** 2021-06-25

**Authors:** Refat Nimer, Samer Swedan, Hassan Kofahi, Omar Khabour

**Affiliations:** 1 Faculty of Applied Medical Sciences, Dept. of Medical Laboratory Sciences, Jordan University of Science and Technology, Irbid 22110, Jordan

## Abstract

**Background:**

The adherence of medical laboratory technicians (MLT) to infection control guidelines is essential for reducing the risk of exposure to infectious agents. This study explored the adherence of MLT towards infection control practices during the COVID-19 pandemic.

**Method:**

The study population consisted of MLT (n = 444) who worked in private and government health sectors in Jordan. A self-reported survey was used to collect data from participants.

**Findings:**

More than 87% of the participants reported adherence to hand-washing guidelines and using personal protective equipment (PPE) when interacting with patients (74.5%), and handling clinical samples (70.0%). Besides, 88.1%, 48.2%, and 7.7% reported wearing of lab coats, face masks, and goggles, at all times, respectively. The majority reported increased adherence to infection control practices during the COVID-19 pandemic. This includes increased PPE use at the workplace (94.2%), increased frequency of disinfection of laboratory surfaces (92.4%) and laboratory equipment (86.7%), and increased frequency of handwashing/use of antiseptics (94.6%). Having a graduate degree was significantly associated with increased adherence of participants to the daily use of goggles/eye protection (p = 0.002), and the use of PPE while handling clinical samples (p = 0.011). Having work experience of >10 years was associated with increased adherence to the use of PPE while handling clinical samples (p = 0.001).

**Conclusion:**

MLT reported very good adherence with most assessed infection control practices. In addition, they reported increased conformity with infection control guidelines during the COVID-19 pandemic.

## Introduction

The emergence of severe acute respiratory syndrome coronavirus 2 (SARS-CoV-2) in 2019, followed by the declaration of the disease (COVID-19) as a pandemic in 2020, had a significant impact on human behavior around the globe [1]. According to the World Health Organization (WHO) a total of 161,513,458 cases and 3,352,109 deaths were reported as of May 15th, 2021 [2]. New public health measures including social distancing and wearing of masks were encouraged or enforced, to slow virus spread and to flatten the infection curve. Since the early days of the pandemic, it was clear that healthcare workers were at higher risk of SARS-CoV-2 infection than the general population [3]. In a recent meta-analysis that included 11 studies, the total proportion of healthcare workers who were positive for the coronavirus among all COVID-19 patients was 10.1% [4]. In addition, the reported mortality rate among healthcare workers was in the range from 0.2% to 0.5% [4,5]. These numbers placed a high burden on the healthcare workers to maintain a safe work environment for themselves and to prevent the transmission of the virus to others.

Infection control deals with the detection and monitoring of factors involved in disease transmission, whether from patient to patient, patients to staff, staff to patients, or among staff members. Based on our experience with SARS-CoV-1, the risk of infection can be reduced significantly by adhering to infection control guidelines, such as the proper use of personal protective equipment (PPE), including masks, goggles/face shield, gloves, and gowns, when necessary, and frequent hand-washing [6,7]. Hand-hygiene was reported as one of the main factors that reduces the risk of SARS-CoV-2 transmission to health care workers [3].

Medical laboratory technicians (MLT) are a vital part of the health care system. They aid in the diagnosis of diseases by performing a wide range of tests on specimens like blood, tissues, urine, stool, and other body fluids [8]. MLT may contract SARS-CoV-2 infection by direct contact with infected patients during sample collection [9]. This is more likely to occur if the duties of the MLT include phlebotomy and sample collection [10]. Collection of clinical samples requires close contact between MLT and the patient, which increases the risk of SARS-CoV-2 transmission from an infected patient to MLT, and vice versa. Furthermore, MLT are in contact with different types of body fluids that may harbor SARS-CoV-2. These fluids may contaminate laboratory surfaces and equipment during sample processing [11,12]. SARS-CoV-2 remains stable on surfaces for hours to days depending on the surface type [13,14]. Contaminated surfaces may lead to infection if a technician touches his eyes, nose, or mouth with the contaminated gloves or before hand-washing/sanitization. This can be prevented by frequent disinfection of the surfaces and regular hand-washing/sanitization [15,16]. Furthermore, some laboratory procedures may generate aerosols or droplets, which may reach the eyes, the mouth, or the nose, or that are inhaled [17,18]. Using face-shield/goggles and masks reduces the risk of infection via this route [19].

Adherence of MLT to infection control guidelines is of the utmost importance to reduce their risk of infection with SARS-CoV-2 [20,21]. The adherence to these guidelines can be influenced by multiple factors including educational level, training on laboratory safety, and years of experience [22]. Therefore, it is essential to assess the adherence of MLT to infection control practices during the pandemic to ensure the safety of medical teams and patients [23,24]. In the current study, the adherence of MLT to infection control practices was evaluated during the pandemic, and the possible independent factors were explored. In addition, the impact of the COVID-19 pandemic on the level of adherence to infection control practices was assessed. The findings of this study should prove helpful to healthcare policymakers and could highlight areas in need of improvement.

## Methods

### Study participants

The study was approved by the institutional review board of Jordan University of Science and Technology (Ref.: 165/132/2020, dated 16/05/2020). This cross-sectional study utilized an online self-administered structured questionnaire in the Arabic language. The study participants were recruited between May 30, 2020, to June 20, 2020, by posting the questionnaire link on several Jordanian MLT groups on Facebook and WhatsApp. Moreover, the link was shared personally to MLT in the contact lists of the investigators. Current workers in Jordanian public or private medical laboratories, including hospitals’ diagnostic laboratories, central referral laboratories, and blood banks were eligible to participate in the study. To ensure anonymity, information regarding participants’ identity and their place of work, were not collected. Informed consent was obtained from each participant. A total of 444 participants completed the questionnaire.

### Study instrument

The questionnaire for this study was designed based on comprehensive literature searches, and the questions were built based on relevant reviewed literature with some modifications. The content and face validations were carried out by a group of experts in the fields of medical laboratories and infectious diseases. The questionnaire was administered using Google forms. It surveyed compliance to key areas of infection control guidelines, including use of personal protective equipment, hand hygiene, cleaning and disinfection of medical equipment and benches, as well as the availability of infection control education.

The questionnaire contained a total of 26 questions divided into three main sections. The first section obtained informed consent from each participant and confirmed the current employment of the participant in a Jordanian medical laboratory. The second section collected general participants’ information including gender, age, highest education, laboratory location (city), and the laboratory section assignment. The third section evaluated participants’ practices on infection control guidelines, and their perceptions of the impact of the COVID-19 pandemic on the level of adherence to these practices. To assess the effect of participants’ education level on adherence to infection control guidelines, the participants were divided into two groups based on their highest educational level; the “Post BSc” group, which included participants with a graduate degree (MSc, or PhD), and the “Up to BSc” group which included participants with a diploma or a BSc degree. Similarly, the effect of work experience on the level of adherence to infection control guidelines was evaluated by dividing the participants into two arbitrary groups: “≤10 years” and “>10 years.”

### Statistical analysis

Analysis of the data was performed using the Statistical Package for the Social Sciences version 23 (IBM Inc., Armonk, New York, United States). The chi-square test was used to compare frequencies. A p-value less than 0.05 was considered statistically significant.

## Results

### Participants’ characteristics

A total of 444 MLT currently working in the government sector (44.6%) and private sector (55.4%), completed the questionnaire. The assigned tasks of the participants were phlebotomy (52.7%), clinical chemistry (50.7%), microbiology (30.8%), serology (26.9%), hematology (24.1%), molecular diagnosis (17.6%), and blood bank (14.6%). ***Table 1*** shows participants’ characteristics in terms of age, gender, highest education, and years of experience. Among the participants, 67.3% were females and 48.9% were ≤30 years old. The highest degrees held by the participants were diploma (13.5%), BSc (61.2%), and MSc/PhD (25.3%). The work experience varied greatly among participants. More than one-third (44.4%) had work experience of five years or less, and 36% had an experience of more than 10 years.

**Table 1:** Demographic characteristics of medical laboratory technicians.


CRITERIA		n	%

Gender	Female	299	67.3

	Male	145	32.7

Age	≤30	217	48.9

	31–40	119	26.8

	>40	108	24.3

Highest education	Diploma	60	13.5

	BSc	272	61.3

	MSc/PhD	112	25.3

Experience (years)	<1	47	10.6

	1–5	150	33.8

	6–10	87	19.6

	>10	160	36.0

Training on infection control	During education	151	34.0

	At workplace	189	42.6

	No training	99	22.3

The workplace has an infection control unit	Yes	258	58.1

	No	186	41.9

Willing to participate in an infection control training workshop	Yes	393	88.5

	No	51	11.5


Infection control training was provided to 42.6% of the participants by their workplace after employment, and to 34.0% before employment by an educational institution (***Table 1***). About one-fourth (22.3%) of the participants reported receiving no training on infection control. Moreover, the majority of the participants (88.5%) expressed willingness to improve their infection control knowledge and skills by agreeing to participate in infection control training workshops (***Table 1***).

### Adherence to infection control guidelines

The adherence of MLT to infection control during the COVID-19 pandemic was evaluated. These include hand washing, the use of PPE, and the frequency of disinfection of objects and surfaces in the work area. The factors that might influence the level of adherence to infection control measures, such as the highest education level and the work experience, were also investigated (***Table 2***).

**Table 2:** Infection control practices of medical laboratory technicians in relation to education level and years of experience.


CRITERIA		TOTAL	EDUCATION LEVEL			WORK EXPERIENCE		
	
			MSC/PHD	BSC/DIPLOMA	P VALUE*	>10 YEARS	≤10 YEARS	P VALUE*
		
		N(%)	N(%)	N(%)		N(%)	N(%)	

**Wash hands before wearing gloves**.	No	203(45.7)	54(46.6)	149(45.4)	0.834	70(43.8)	133(46.8)	0.532

	Yes	241(54.3)	62(53.4)	179(54.6)		90(56.3)	151(53.2)	

**Wash hands after removing gloves**.	No	56(12.6)	13(11.2)	43(13.1)	0.596	22(13.8)	34(12.0)	0.588

	Yes	388(87.4)	103(88.8)	285(86.9)		138(86.3)	250(88.0)	

**Wash hands before leaving the workplace**.	No	45(10.1)	8(6.9)	37(11.3)	0.179	17(10.6)	28(9.9)	0.797

	Yes	399(89.9)	108(93.1)	291(88.7)		143(89.4)	256(90.1)	

**Use of gloves during lab work**.	No	12(2.7)	3(2.6)	9(2.7)	0.654	4(2.5)	8(2.8)	0.975

	Yes	432(97.3)	113(97.4)	329(97.3)		165(97.5)	276(97.2)	

**Gloves removed when leaving the lab room**.	No	10(2.3)	3(2.6)	7(2.1)	0.482	5(3.2)	5(1.8)	0.263

	Yes	434(97.7)	113(97.4)	321(97.9)		155(96.9)	279(98.2)	

**Daily use of Lab coat**.	No	16(3.6)	4(3.4)	12(3.7)	0.871	6(3.8)	10(3.5)	0.963

	Yes	428(96.4)	112(96.6)	316(96.3)		154(96.2)	274(76.5)	

**Daily use of goggles/eye protection**.	No	302(68.0)	64(55.2)	238(72.6)	**0.002**	98(61.3)	204(71.8)	0.072

	Yes	142(32.0)	52(44.8)	90(27.4)		63(38.7)	80(28.2)	

**Daily use of face mask**.	No	68(15.3)	17(14.7)	51(15.5)	0.799	26(16.3)	42(14.8)	0.479

	Yes	376(84.7)	99(85.3)	177(84.5)		134(83.7)	242(85.2)	

**PPE used when interacting with patients**.	No	113(25.5)	31(26.7)	82(25.0)	0.714	46(28.7)	67(23.6)	0.231

	Yes	331(74.5)	85(73.3)	246(75.0)		114(71.3)	217(76.4)	

**PPE used when handling clinical samples**.	No	133(30.0)	24(20.7)	109(33.2)	**0.011**	33(20.6)	100(35.2)	**0.001**

	Yes	311(70.0)	92(79.3)	219(66.8)		127(79.4)	184(64.8)	


The vast majority of participants reported washing their hands before and after using gloves (n = 388; 87.4% and 395; 89.0% respectively), and before leaving the workplace (n = 399; 89.9%). The majority reported using PPE when interacting with patients (74.5%) and handling clinical samples (70.0%). Moreover, 88.1%, 48.2%, and 7.7% reported wearing of lab coats, face masks, and goggles, at all times, respectively (***Table 2***).

Having a post-BSc graduate degree was significantly associated with increased adherence of participants to the daily use of goggles/eye protection (p = 0.002), and the use of PPE while handling clinical samples (p = 0.011). Having work experience of 10 years or more was significantly associated with increased adherence to the use of PPE while handling clinical samples (p = 0.001, ***Table 2***).

Finally, changes in the level of adherence to infection control practices due to the COVID-19 pandemic as perceived by the participants were investigated (***Table 3***). The majority of the participants reported a positive impact on adherence to infection control practices at the workplace. For example, the majority of the participants reported that the pandemic led to increased PPE use at the workplace (94.2%), increased frequency of disinfection of laboratory surfaces (92.4%) and laboratory equipment (86.7%), and increased frequency of handwashing/use of antiseptics (94.6%).

**Table 3:** Changes in the practices of medical laboratory technicians regarding infection control during the COVID-19 pandemic.


CRITERIA/CHANGE IN THE PRACTICE DURING COVID-19	INCREASED N(%)	NO CHANGE N(%)

Use of PPE.	418(94.1)	26(5.9)

Frequency of disinfection of laboratory surfaces.	410(92.3)	34(7.7)

Frequency of disinfection of laboratory equipment.	385(86.7)	59(13.3)

Frequency of hand washing/use of antiseptics.	420(94.6)	24(5.4)


## Discussion

This study investigated the level of self-reported adherence of MLT to infection control practices during the COVID-19 pandemic, the factors that might influence adherence, and the perceptions of MLT on the impact of the pandemic on adherence. MLT reported very good adherence with most assessed infection control practices. In addition, they reported increased conformity with infection control guidelines during the COVID-19 pandemic.

The participants reported compliance with handwashing in some of the queried conditions. The majority of respondents reported washing their hands after removing gloves, and before leaving the workplace. On the other hand, about half reported low adherence to handwashing before wearing gloves. Hence, it is necessary to improve MLT adherence to handwashing. This might be achieved by offering periodical post-employment infection control courses/workshops. Poster installation in the laboratory to remind MLT of hand-hygiene policies was reported to help increase compliance to the “no-jewelry” policy [25]. Hence, the use of posters may also help in improving adherence to handwashing at the laboratory setting. For comparison, hand hygiene was observed by laboratory staff at rates of 71.4% and 68.8% in studies from Kenya and Australia, respectively [26,27].

Most participants reported using gloves or lab coats on a daily basis. In contrast, the adherence to the daily use of goggles/eye protection was relatively low. Goggles/eye protection use is necessary to protect MLT while performing procedures that potentially generate splashes or sprays from clinical samples. Not all MLT are performing procedures that potentially generate splashes or sprays, which might partly explain this finding. In addition, goggles/eye protection may not be available or is available in limited quantities in many Jordanian medical laboratories. On the other hand, some MLT may not be aware of the importance of using these PPE. Compliance with the use of these PPE is crucial during the COVID-19 pandemic, since droplets and aerosols are considered major routes of transmission [28]. For comparison, use of gloves and other PPE was observed by laboratory staff at rates of 78% and 71.4% in a study from Kenya [26], and 100% in a study from Nigeria [29].

The majority of MLT reported using masks either all or some of the time. Mask use is not a routine infection control practice for MLT under normal circumstances, especially for those who are not in direct contact with patients. However, adherence to this practice in the laboratory setting became mandatory in many countries as a result of the COVID-19 pandemic [30].

As expected, the COVID-19 pandemic had a positive impact on the level of compliance of the majority of MLT with infection control practices. Using PPE at the workplace, the frequency of disinfection of laboratory surfaces, and the frequency of handwashing/use of antiseptics increased dramatically as a result of the pandemic. A similar finding was reported from Saudi Arabia [31]. Participants also reported adherence to other practices that reduce the risk of disease transmission at the laboratory setting, such as ensuring the wearing of masks by laboratory patrons and limiting patient’s companions to one person, among others. Additional practices and strict precautions must be utilized in the event of new epidemics or pandemics. We hypothesize that MLT dealing with patients and specimens will observe increased compliance and application of infection control guidelines in the following months. This is based on the following: our still-incomplete knowledge about COVID-19, the absence of effective COVID-19 treatment, and the relatively long period required to complete vaccine rollout in Jordan and worldwide [32].

Disinfection of equipment and surfaces is hypothesized to be an essential infection control practice to prevent SARS-CoV-2 transmission, as the virus may remain stable on surfaces for several days [14]. Consequently, the vast majority of MLT reported an increased frequency of disinfection of surfaces and equipment at the workplace as a result of the COVID-19 pandemic. This behavior reduces the risk of transmission of SARS-CoV-2 and other pathogens. It may also improve the quality of laboratory test results by reducing the risk of contamination, especially in the microbiology and molecular biology laboratories. This behavior change will likely have a positive impact on infection control and the quality of results even after the end of the COVID-19 pandemic.

Having a post-BSc graduate degree was associated with increased adherence of MLT to several infection control guidelines, including the daily use of eye protection, and the use of PPE when handling clinical samples. These results are consistent with the fact that a higher level of education increases knowledge and likely compliance with infection control guidelines [33].

Having work experience of more than 10 years was significantly associated with increased adherence to using PPE when handling clinical samples. These results are in agreement with those reported by Slyne et al., where the experience was demonstrated to enhance adherence to infection control practices [34].

Less than one-fourth of the participants reported not receiving any education or training on infection control. This could be attributed to lack of training during employment or suboptimal study curricula. The deficiency will probably increase the likelihood of not utilizing proper infection control measures in the laboratory setting. This deficiency should be remedied by qualified governmental infection control teams that implement and promote infection control practices [35]. This might include the implementation of a specific certificate and mandating continuing education for infection prevention and control professionals [36]. In addition, integration of infection prevention and control practices in the curriculum of undergraduate medical laboratory courses might play an important role in the promotion of related knowledge, skills and attitudes [37]. Moreover, utilization of “Train-the-Trainers” approach in hand hygiene was shown to be effective in enhancing knowledge and sharing experiences among healthcare professionals [38]. Having satisfactory education, training and knowledge on infection control by MLT is essential, not only due to the constant potential exposure to hazards in the laboratory setting, but also for coping with the long-term persistence of COVID-19, and future epidemics and pandemics [39].

It is suggested that enhancing public awareness of infectious agents and the importance of basic hygiene can significantly limit spread of infectious diseases in the community [40]. Thus, infection control and prevention should be extended beyond the health setting to include all areas of public health. Among suggested approaches of public health for control and prevention of infectious disease are implementing vaccinations programs, management of sources of infection [41], education of the public to promote good practices that reduce the spread of infectious agents, and supporting and coordinating community efforts aimed at protecting society from infectious diseases [42].

Although this study provided insights into the level of adherence of MLT in Jordan to infection control guidelines during the COVID-19 pandemic, it had some limitations. This was a cross-sectional survey performed via an anonymous questionnaire, so the results presented were self-reported partly based on the participants’ reliability, honesty, and ability to recall. It is likely that some of the participants responded to the questions in a manner that they believe to be objectively appropriate rather than entirely correct or may have provided more extreme responses based on personal beliefs. Also, some responses may be prone to recall bias. Thus, the study findings should be confirmed by direct assessment of infection control practices in the actual laboratory setting and the prevalence of occupational infection/injury reported in the laboratory documentations.

## Conclusions

Overall, Jordanian MLT demonstrated good compliance with most of the assessed infection control practices during the COVID-19 pandemic. MLT reported that the COVID-19 pandemic led to increased rates of compliance with infection control practices compared to that before the pandemic. However, compliance to some practices, such as using goggles/eye protection, require further improvements.

The adherence of MLT to infection control guidelines can be improved by providing continuing education programs on infection control and by monitoring adherence to these guidelines by an infection control committee (when present) or the laboratory manager. It is also crucial that medical laboratories are provided with a sufficient supply of PPE. These measures will improve the ability of MLT to deal with the current COVID-19 pandemic and future outbreaks.
